# Dengue NS1 as a Driver of Immune-Mediated Pathogenesis

**DOI:** 10.3390/tropicalmed11050128

**Published:** 2026-05-08

**Authors:** Upeksha S. Wanigarathna, Senaka Rajapakse, Sisira L. Pathirana, Shiroma M. Handunnetti, Andreas Nitsche, Narmada Fernando

**Affiliations:** 1 Institute of Biochemistry, Molecular Biology and Biotechnology, University of Colombo, Colombo 00300, Western Province, Sri Lanka; 2 Department of Clinical Medicine, Faculty of Medicine, University of Colombo, Colombo 00700, Western Province, Sri Lanka; 3 Highly Pathogenic Viruses Centre for Biological Threats and Special Pathogens, Robert Koch Institute, 13353 Berlin, Germany

**Keywords:** DENV, dengue, NS1, immunopathogenesis, endothelial dysfunction, endothelial disruption, permeability, complement, anti-NS1, immune cells, lipoprotein

## Abstract

Dengue infection remains a major global health concern, with a subset of patients progressing from self-limited dengue fever to severe disease characterised by plasma leakage, shock, and organ dysfunction. The dengue non-structural protein 1 (NS1), a multifunctional glycoprotein expressed on infected cells and secreted into circulation, has emerged as a key mediator linking viral infection to immune-driven vascular pathology. This review synthesises experimental, animal, and human clinical evidence on NS1-driven immunopathogenesis, focusing on mechanisms leading to endothelial dysfunction and increased vascular permeability. NS1 modulates the complement system in a context-dependent manner, contributing to immune evasion by inhibiting terminal complement complex formation, while also promoting antibody-dependent complement activation associated with severe disease. Additionally, NS1 directly disrupts endothelial barrier integrity through disruption of adherens and tight junction architecture, Ang-2/Tie2 imbalance, activation of RhoA/ROCK (RhoA/Rho-associated coiled-coil-containing protein kinase) signalling, and enzymatic degradation of the endothelial glycocalyx, with further amplification through inflammatory mediators. In addition, evidence shows that NS1 activates innate immune signalling, perturbs platelet biology and haemostasis, and forms pro-inflammatory complexes with lipoproteins. Moreover, anti-NS1 antibodies may be both protective and pathogenic. Collectively, these data position NS1-linked pathways as rational targets for adjunctive therapies and next-generation vaccines aimed at preventing vascular leakage and severe dengue infection.

## 1. Introduction

Dengue virus (DENV) infection remains a major and expanding vector-borne threat in tropical and subtropical regions and is caused by four antigenically related but genetically distinct serotypes (DENV-1 to DENV-4) [1,2,3,4]. While most infections are asymptomatic or self-limiting, a clinically important minority progresses to severe dengue infections, including dengue haemorrhagic fever and dengue shock syndrome [1,4]. A defining biological event in severe disease is systemic plasma leakage across the vascular endothelium, which can precipitate intravascular volume depletion, hypovolaemic shock, and organ dysfunction [5,6].

The non-structural protein 1 (NS1) has emerged as a central mediator linking viral infection to immune-driven vascular pathology. NS1 is a multifunctional glycoprotein that is expressed on the surface of infected cells and, uniquely among DENV proteins, is also released into the circulation as soluble NS1 (sNS1) [7]. Accumulating experimental and clinical evidence indicates that sNS1 contributes to pathogenesis through several convergent mechanisms. It has been shown to modulate complement and thereby shape both immune evasion and inflammatory injury [8]; to directly disrupt endothelial barrier function and promote vascular leak in vivo and in vitro [9]; and to activate innate immune signalling pathways that amplify cytokine and chemokine responses [10]. In parallel, NS1-driven effects have been linked to platelet activation and haemostatic perturbation, providing a plausible bridge between inflammation, thrombocytopenia, and microvascular dysfunction in severe dengue infection [11].

This review synthesises current evidence supporting NS1 as a driver of immune-mediated pathogenesis in DENV, with particular emphasis on mechanistic links between NS1 innate immune activation, including complement cascade, endothelial dysfunction and plasma leakage, and antibody-dependent effects that may influence progression from uncomplicated illness to life-threatening disease.

## 2. Search Strategy

A literature search was conducted in PubMed/MEDLINE and Google Scholar from database inception to 2025 to identify studies evaluating the role of DENV NS1 in dengue-related immunopathogenesis. The search combined controlled vocabulary and free-text terms related to dengue, NS1, and immunopathogenesis using Boolean operators: (“dengue” OR “dengue virus” OR “DENV”) AND (“nonstructural protein 1” OR “NS1”) AND (“endothelium” OR “endothelial cell” OR “vascular permeability” OR “vascular leak” OR “plasma leakage” OR “pathogenesis” OR “immunopathogenesis”) and, where appropriate, (‘immune cells” OR “macrophages” OR “cytokines” OR “complement” OR “Toll-like receptor 4” OR “TLR4” OR “antibody” OR “anti-NS1” OR “endothelial glycocalyx”). No study design filters were applied, and in vitro, animal and human clinical studies were considered. Reference lists of key primary articles and relevant reviews were searched manually to identify additional publications. We included original studies that reported experimental or clinical data. Review articles, conference abstracts without sufficient data, studies without NS1-related outcomes, and non-English publications were excluded, although reviews were used to identify additional primary data sources.

## 3. Effect of DENV NS1 on Immunopathogenesis

### 3.1. Effects on the Complement System

DENV NS1 has multiple context-dependent effects on complement, spanning immune evasion and complement-driven immunopathology. Complement normally restricts infection upstream, through opsonisation and neutralisation via C3b deposition, and downstream through formation of the membrane attack complex (MAC). Experimental studies indicate that NS1 can both attenuate productive complement activity on virus and infected cells and, under certain conditions, promote complement activation associated with severe disease manifestations [7,8].

A key immune evasion mechanism involves interference with early steps shared by the classical and lectin pathways. NS1 binds C4 and C4b and forms a functional complex with C1s/proC1s, enhancing cleavage of C4 in plasma and thereby depleting intact C4 available for deposition on virions or infected-cell surfaces. This non-productive complement activation reduces C4b deposition and downstream C3 convertase activity, leading to diminished C3b opsonisation and reduced complement-mediated neutralisation. Importantly, NS1 does not appear to inhibit C4 activation by accelerating C3 convertase decay or acting as a factor I cofactor; rather, it promotes C4 activation away from target surfaces through C1s protease, where nascent C4b is rapidly inactivated. This strategy may be particularly effective in facilitating viral spread within peripheral tissues where complement concentrations are lower and sNS1 may accumulate [8].

In secondary heterologous infection, NS1 may shift from primarily facilitating immune evasion to contributing to pathology through antibody-dependent activation of complement. Pre-existing anti-NS1 antibodies form immune complexes with circulating NS1, recruiting complement and promoting terminal pathway activation at endothelial surfaces, potentially increasing C5b-9 generation and contributing to vascular leak in severe dengue infection [8].

NS1 can also inhibit terminal pathway effector function by targeting regulators and components involved in MAC assembly. NS1 binds the terminal complement regulator vitronectin, and NS1–vitronectin complexes are detectable in plasma from dengue patients. In functional assays, NS1 reduces MAC-mediated lysis, consistent with suppression of terminal pathway killing. Mechanistically, NS1 interacts with multiple terminal pathway proteins (including C5, C6, C7, and C9) and inhibits C9 polymerisation, a critical step in MAC pore formation; this effect is enhanced in the presence of vitronectin, a multifunctional glycoprotein. Through these mechanisms, DENV NS1 alters complement activation within the host [12]. Alongside these evasion mechanisms, NS1 also shows the ability to activate complement in ways linked to disease severity. In vitro, sNS1 derived from DENV-infected Vero cells supernatants or recombinant sNS1 from 293T cells were shown to trigger complement activation up to the level of the terminal pathway, with increased generation of SC5b-9; these effects were amplified in the presence of anti-NS1 antibodies, consistent with immune complex-mediated activation. Membrane-associated NS1 on infected or NS1-expressing cells likewise promotes antibody-dependent complement deposition, including C3 fragments and C5b-9 on cell surfaces. Clinically, NS1 and SC5b-9 were shown to be higher in DHF/DSS than in dengue fever and were not detected in non-dengue febrile controls. Paired sampling in DSS showed higher NS1 and complement activation products (including C5a and SC5b-9) in pleural fluid than in plasma, supporting localised complement activation at sites of plasma leakage [7].

Finally, NS1 interacts with clusterin, a soluble regulator that limits MAC assembly. Clusterin was identified as a prominent NS1-binding protein in human plasma by affinity purification-mass spectrometry, with the interaction confirmed by Western blotting. Binding to clusterin was also demonstrated with recombinant sNS1 released from 293T cells and sNS1 from DENV-infected kidney epithelial cells derived from the African green monkey (Vero cells). Based on these findings, NS1-clusterin binding could weaken the function of clusterin in the terminal pathway, either by reducing functional clusterin availability or interfering with its role during MAC assembly, thereby permitting greater complement-mediated damage of infected cells and contributing to severe disease [13].

**Figure 1:**
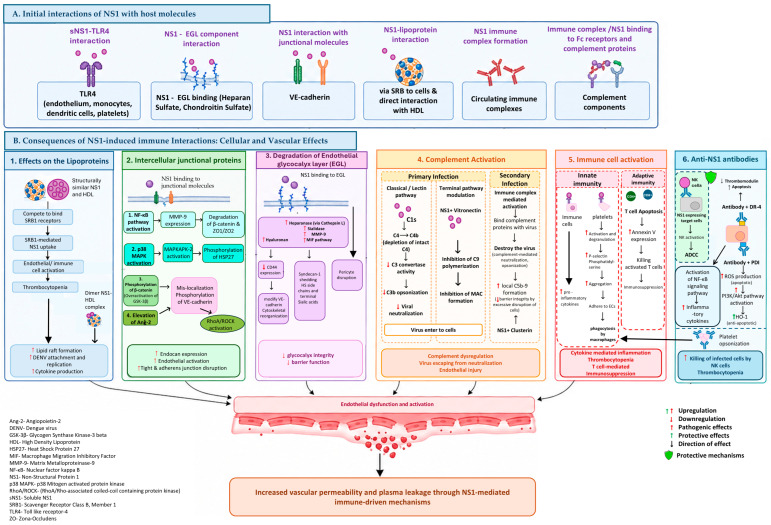
NS1 interactions with host receptors and cellular targets and their immunological consequences. (**A**) Initial interactions of NS1 with host molecules. (**B**) Consequences of NS1-induced immune interactions. (**1**) NS1 binds directly with HDL or via SRB-1 receptors, initiating pathological processes such as cytokine production and thrombocytopenia, which contribute to increased vascular permeability. (**2**) NS1 interaction with intercellular junctional proteins activates signalling pathways, leading to disruption of junctional integrity. (**3**) NS1 binding to EGL components activates degradative enzymes, facilitating the breakdown of EGL-associated proteins. (**4**) NS1-mediated complement activation may enable viral evasion of complement-mediated neutralisation, while excessive terminal attack complex formation contributes to cellular damage during secondary infection. (**5**) Direct binding of NS1 to TLR4 induces inflammatory cytokine production and NS1-TLR4 interactions on platelets promote phagocytosis by macrophages, leading to thrombocytopenia. (**6**) Anti-NS1 antibodies exhibit both pathogenic and protective roles. Pathogenic effects include ROS production leading to cell apoptosis and platelet opsonization, resulting in macrophage-mediated phagocytosis and thrombocytopenia. Protective effects involve selective ADCC against NS1-expressing target cells mediated by NK cells.

Taken together, NS1 shapes complement responses along two axes: (i) early immune evasion through diversion of C4 away from target surfaces and suppression of terminal pathway pore formation, and (ii) complement activation and generation of terminal complex mediators that correlate with severe dengue infection (Figure 1).

### 3.2. Direct Effect of DENV NS1 on Endothelial Disruption

#### 3.2.1. Disruption of Intercellular Junctional Proteins

The endothelial barrier is maintained by coordinated regulation of intercellular junction, including tight junction proteins (claudins, occludins, ZO 1/2) and adherens junction complexes (VE-cadherin, catenins), which regulate paracellular flux. DENV NS1 triggers vascular leakage by attacking these structures through multiple interconnected signalling pathways [14].

sNS1 can directly remodel adherens junction architecture in a tissue-dependent manner. In endothelial monolayers, NS1 induces mis-localisation of VE-cadherin and β-catenin away from cellular junctions, indicating junctional disassembly, without clear evidence of major early reductions in total protein abundance. This mechanism is accompanied by evidence of clathrin-mediated internalisation of VE-cadherin and increased β-catenin phosphorylation through the glycogen synthase kinase-3 beta (GSK-3β) pathway, which destabilises the VE-cadherin/β-catenin complex and increases endothelial permeability. Importantly, pharmacologic inhibition of GSK-3β using a peptide inhibitor restores barrier integrity in vitro in human umbilical vein endothelial cell (HUVEC) and human brain microvascular endothelial cell (HBMEC) models and attenuates NS1-driven leakage in mouse dermal leakage assays in flaviviruses, including DENV, providing functional support for this signalling pathway in NS1-induced hyperpermeability [15].

NS1 also weakens junctional stability by shifting endothelial signalling toward a Tie2-destabilising state. In human dermal microvascular endothelial cells (HMECs), NS1 increases permeability in parallel with angiopoietin-2 (Ang-2) release, VE-cadherin phosphorylation and loss from junctions, and activation of RhoA/ROCK signalling, consistent with increased cytoskeletal contractility and junctional disassembly. Barrier dysfunction is largely reversible by restoring stabilising signalling. Recombinant Ang-1 (a physiological antagonist of Ang-2) prevents Ang-2 release and downstream VE-cadherin internalisation, while blockade of Tie2 signalling or inhibition of Rho/ROCK similarly prevents the increase in permeability. Accordingly, NS1-related adherens junction destabilisation appears to be mediated via Ang-2/Tie2 imbalance and RhoA/ROCK activation [16].

Beyond junction remodelling, NS1 can promote an activated endothelial phenotype that may prime the barrier for dysfunction. Recombinant NS1 from multiple serotypes increases endocan expression and secretion in the human microvascular endothelial cell lines (HMEC)-1 and the human umbilical vein cell line (EA. hy926), and this response is attenuated by TLR4 (Toll-like receptor 4) blockade, showing that NS1 causes endocan production through TLR4 signalling. Clinically, serum endocan levels are elevated in dengue patients compared with healthy controls during the early phase of infection and are higher in NS1 antigen-positive than NS1-negative patients, although endocan levels do not clearly distinguish cases with and without warning signs. NS1 was assessed qualitatively and downstream TLR4 pathway activation has not been directly demonstrated. Taken together, these findings link circulating NS1 to endothelial activation and suggest a plausible route by which inflammatory signalling can intersect with junctional stability [17].

NS1 can additionally amplify barrier dysfunction indirectly. In severe dengue infection, circulating matrix metalloproteinase-9 (MMP-9) levels increase alongside NS1 and correlate with NS1 detection. Mechanistically, NS1 enhances MMP-9 expression and secretion predominantly in peripheral blood mononuclear cells (PBMCs)/macrophages via NF-κB (nuclear factor kappa B) activation and increases MMP-9 proteolytic activity by pro-MMP-9 maturation through direct interaction. At the endothelial interface, NS1 together with MMP-9 reduces barrier function and is associated with loss of junctional proteins, including β-catenin and ZO-1/2, with inhibitor experiments indicating MMP-9 dependence. In vivo, both pharmacologic MMP-9 inhibition and genetic MMP-9 deficiency reduce NS1-associated vascular leakage [18].

In vitro, NS1 activates endothelial stress signalling pathways, particularly p38 mitogen-activated protein kinase (p38 MAPK), leading to phosphorylation of downstream effectors such as MK2 and heat shock protein 27 (HSP27) in HUVECs. This signalling cascade is associated with a rapid but reversible disruption in barrier integrity, as measured by trans-endothelial electrical resistance (TEER). Chemical inhibition of p38 MAPK restores barrier function, supporting its role in NS1-induced permeability changes [19]. Collectively, these studies support a layered, convergent model of NS1-driven vascular leak (Figure 1). Clinical biomarker patterns are consistent with combined glycocalyx injury and junctional involvement [14], while mechanistic work shows that sNS1 can directly destabilise adherens junction organisation through VE-cadherin internalisation, β-catenin phosphorylation (GSK-3β-linked) [15] and Ang-2/Tie2 imbalance with downstream RhoA/ROCK activation [16]. In parallel, NS1 can prime or amplify dysfunction via TLR4-associated endothelial activation [17] and by inducing MMP-9 in immune cells, which then targets junctional substrates to drive more durable barrier breakdown, with strong in vivo support from inhibitor and knockout approaches [18]. Rather than acting through isolated pathways, these mechanisms plausibly reinforce one another, steering the endothelium toward a common endpoint of junctional disorganisation, increased paracellular permeability, and plasma leakage in severe dengue infection.

#### 3.2.2. Endothelial Glycocalyx (EGL) Disruption

Endothelial barrier function, including the integrity of the transcellular pathway, is critically influenced by the endothelial glycocalyx layer (EGL), a negatively charged luminal meshwork enriched in proteoglycans (syndecan-1) and glycosaminoglycans (GAGs) such as chondroitin sulphate, heparan sulphate, and hyaluronan. In dengue infection, circulating markers consistent with EGL shedding, particularly syndecan-1 and chondroitin sulphate, are elevated in patients with plasma leakage and increase with leak severity, supporting clinically relevant glycocalyx damage during the critical phase [14].

A mechanistic basis for EGL targeting is the ability of sNS1 to bind back onto uninfected tissues via electrostatic interactions with highly sulphated GAGs, with strong dependence on heparan sulphate and chondroitin sulphate E. Binding is cell type-selective, prominent on epithelial/mesenchymal cells and certain ECs, but minimal on primary hematopoietic cells. Ex vivo tissue experiments on mouse and human tissue cryosections demonstrate rapid NS1 deposition on microvascular endothelium and serosal surfaces (lung/peritoneum), which are clinically relevant sites of fluid accumulation [20]. These findings help explain the organ-specific vascular leakage observed in severe disease. In vitro studies support a direct endothelial cell-intrinsic mechanism in which NS1 engages enzymatic pathways that dismantle the EGL. In microvascular endothelial monolayers, such as human pulmonary microvascular endothelial cells (HPMECs), human dermal microvascular endothelial cells (HMEC-1) and HUVECs, recombinant NS1 induces time- and dose-dependent loss of surface sialic acids (tracked by lectin staining), together with upregulation of sialidases (neuraminidases), consistent with enzymatic trimming of terminal glycans. In parallel, NS1 increases cathepsin L activity and promotes proteolytic processing of pro-heparanase into active heparanase, enabling cleavage of heparan sulphate chains and promoting shedding of syndecan-1-rich EGL structures. Pharmacologic blockade of neuraminidases, cathepsin L, or heparanase substantially attenuates NS1-induced hyperpermeability. In vivo leakage assays further support glycocalyx degradation as an early and targetable component of NS1-mediated hyperpermeability [21,22]. Data of infected murine models show increased circulating sialic acid alongside high NS1 during severe/lethal infection, consistent with systemic glycan disruption in vivo [23].

Patient data confirm that the heparanase pathway is activated during acute disease: plasma heparanase activity and circulating glycocalyx breakdown markers, including heparan sulphate and syndecan-1 are elevated in acute dengue infection and normalise during recovery, with correlations to clinical features of plasma leakage. An inverse relationship between heparanase activity and platelet count suggests platelet involvement in heparanase dynamics. However, ex vivo experiments using healthy donor platelets indicate that thrombin is a strong driver of platelet heparanase release and activity in a platelet-number-dependent manner, whereas recombinant DENV2 NS1 alone does not trigger platelet activation markers such as P-selectin, LAMP-1 or heparanase release. Live DENV2 can induce heparanase release, but interpretation of this finding is complicated by platelet loss/aggregation. Collectively, these findings suggest that, in patients, coagulation-associated platelet activation may contribute to circulating heparanase independently of direct NS1 platelet activation, while NS1-driven heparanase responses are more consistently supported through endothelial and inflammatory pathways [24].

A major amplification pathway links NS1 to glycocalyx degradation via macrophage migration inhibitory factor (MIF). In sera from acute dengue patients, NS1, MIF, heparanase-1 (HPA-1), MMP-9, and syndecan-1 (CD138) are elevated compared with controls; CD138 and MIF are highest in severe infection, while MMP-9 rises most clearly in dengue infection with warning signs rather than in severe infection, suggesting timing/phase dependence. In severe dengue infection, CD138 correlates positively with NS1 and MIF, whereas HPA-1 and MMP-9 do not show a similar positive association, consistent with NS1/MIF signalling tracking with glycocalyx shedding. NS1 also promotes autophagy by p62 degradation and conversion of LC3-I to LC3-II. Both MIF and autophagy formation are involved in NS1-induced vascular leakage [25]. Mechanistically, recombinant NS1 induces MIF release, increases active HPA-1, and promotes CD138 shedding together with increased permeability in endothelial systems. In parallel, NS1 stimulates leukocytes to secrete MMP-9 (validated by protein quantification and gelatine zymography), and amplify endothelial leak and CD138 shedding in an MMP-9-dependent manner. Across in vitro and in vivo models, MIF inhibition or neutralisation reduces HPA-1/MMP-9 induction, glycocalyx shedding, and leakage. Related work also shows NS1-triggered MIF release coupled to endothelial autophagy and barrier dysfunction. This suggests another downstream NS1–MIF pathway that destabilises barrier structure under high-NS1 conditions [23,26].

NS1 also modulates hyaluronan within the EGL and its signalling interface with the endothelium. High NS1 level correlate with elevated serum hyaluronan levels. NS1 exposure decreases CD44 expression in ECs, thereby affecting the integrity of blood vessels. Hyaluronan synthesis in dermal fibroblasts and ECs promote inflammation. In cultured ECs, hyaluronan–CD44 interactions enhance endothelial permeability and disrupt NS1-induced endothelial integrity by modifying VE-cadherin and cytoskeleton reorganisation [27]. In vitro, NS1 induces hyaluronan synthesis programmes in endothelial cells and dermal fibroblasts, and these effects are amplified by inflammatory cytokines [27].

Finally, NS1 can amplify microvascular leaks by disrupting perivascular support. When pericytes are co-cultured with primary human vascular cells (HUVECs and SVP-saphenous vein pericytes), NS1 causes pericyte/mural cell dysfunction and increases endothelial hyperpermeability. NS1 impairs pericyte-mediated stabilisation of endothelial network structures in 3D microvascular assays. These effects occur without major changes in pericyte viability, growth, or migration and can also be observed in non-contact co-cultures, suggesting a predominantly paracrine mechanism. Although NS1 does not directly cleave the endothelial glycocalyx through pericytes, impaired pericyte support plausibly lowers barrier integrity and amplifies the impact of EGL disruption, thereby worsening NS1-driven hyperpermeability in severe dengue infection [28].

Taken together, the literature supports a convergent model in which NS1 promotes EGL disruption through cooperating steps (Figure 1): (i) GAG-dependent docking concentrates NS1 on susceptible microvascular beds; (ii) NS1 activates endothelial sheddase programmes (neuraminidases and cathepsin L-dependent heparanase activation) that directly dismantle EGL glycans and proteoglycan scaffolds, with supportive murine glycan signals; (iii) an NS1–MIF inflammatory relay amplifies heparanase- and MMP-9-linked shedding across endothelial and leukocyte compartments, aligned with MIF-linked barrier dysfunction; (iv) host physiological modifiers, including thrombin-driven platelet heparanase, may contribute to circulating heparanase during acute illness without requiring direct platelet activation by NS1; and (v) EGL remodelling feeds forward into sustained hyperpermeability via hyaluronan–CD44 signalling, junctional/cytoskeletal reorganisation, and impaired pericyte stabilisation of the microvascular unit. This integrated framework aligns with clinical biomarker signatures linking syndecan-1 and chondroitin sulphate to leakage severity and supports EGL disruption as an early and targetable determinant of dengue-related vascular leak.

### 3.3. NS1-Mediated Immune Cell Activation

#### 3.3.1. DENV NS1 Functions on Innate Immune Cells

Innate immune cells detect infection via pattern-recognition receptors (PRRs), which recognise conserved pathogen-associated molecular patterns (PAMPs). sNS1 behaves as a viral PAMP, engaging TLR4-dependent signalling to activate monocytes/macrophages and drive inflammatory mediator release in a manner that functionally resembles lipopolysaccharide (LPS)-driven innate activation. In vitro, NS1 stimulates cytokine and chemokine production from macrophage and PBMC preparations, including TNF-α and IL-6, and TLR4 blockade or inhibition attenuates these responses, supporting a dominant role for TLR4 in NS1-induced innate activation [10,29]. Importantly, the interpretation of PRR-recognised NS1 depends on the source and purity of NS1 preparation; earlier suggestions of TLR2/6 involvement appear most consistent with preparation-related confounding. Later experiments using low-endotoxin, insect-derived NS1 and PRR-deficient macrophages support TLR4 dependence without a requirement for TLR2 or TLR6, underscoring the need for stringent endotoxin and contaminant controls in NS1–PRR studies [29].

In patient cohorts with serial sampling, circulating NS1 levels correlate positively with serum IL-10 throughout the illness course, and ex vivo stimulation experiments support a direct capacity of bacteria, and mammalian expressed recombinant NS1 to induce IL-10 production in monocytes, consistent with NS1 shaping both inflammatory and counter-regulatory innate responses [30].

NS1-mediated innate activation is also connected to vascular pathology in vivo. In interferon receptor-deficient mice, purified hexameric NS1 can trigger vascular leak in the absence of viral infection in a dose-dependent manner and results in leakage in the lungs, liver, spleen, and intestines, similar to that observed in severe dengue infections in human. Combining it with sublethal DENV2 challenge worsens outcomes alongside increased systemic inflammatory cytokines, including TNF-α and IL-6. NS1 has also been reported to induce dose- and time-dependent endothelial barrier dysfunction in HPMECs and primary HUVECs in vitro [9]. Additionally, recombinant hexameric NS1 from multiple serotypes has been shown to activate human platelets largely through TLR4 signalling, increasing markers of platelet activation (e.g., P-selectin/CD62P upregulation and phosphatidylserine exposure) and sensitising platelets to aggregation at subthreshold adenosine diphosphate (ADP). Activated platelets show enhanced adhesion to endothelial monolayers and increased uptake by macrophage-lineage cells, and in vivo models support NS1–TLR4-dependent contributions to pro-inflammatory cytokine production, thrombocytopenia and haemorrhagic manifestations, consistent with a pathway in which NS1 promotes platelet activation and clearance while amplifying vascular leakage [11].

Finally, NS1 can modulate antigen-presenting innate cells in ways that amplify downstream inflammation during infection. Purified extracellular DENV1 NS1 is taken up by human monocyte-derived dendritic cells in a dose- and time-dependent manner, and NS1 pre-exposure increases the susceptibility to DENV infection while enhancing early viral RNA replication. Following infection, NS1-primed dendritic cells produce increased pro-inflammatory cytokines and chemokines, including IL-6, TNF-α, CCL2, and CXCL10, supporting NS1-primed dendritic cells in amplifying inflammatory signalling and immune cell recruitment during dengue infection [31].

Collectively, these studies support an integrated model in which NS1 acts as a circulating innate immune activator that engages TLR4-linked activation in monocyte/macrophage and platelets, induces both pro-inflammatory and regulatory responses, and primes dendritic cells for enhanced infection and inflammation, ultimately contributing to endothelial dysfunction, thrombocytopenia, and plasma leakage (Figure 1).

#### 3.3.2. DENV NS1 Function on Adaptive Immune Cells

Exposure to recombinant DENV NS1 is associated with increased T-cell apoptosis signalling (Figure 1). In patients sampled serially, circulating NS1 levels correlated positively with annexin V expression on CD3^+^ T cells during acute infection, consistent with greater apoptosis in vivo. Ex vivo, incubation of PBMCs from healthy dengue-seropositive donors with NS1 (250–500 ng/mL) produced a dose-related increase in annexin V staining in both CD4^+^ and CD8^+^ T cells, although the effect varied between individuals and was not statistically significant. Notably, NS1 levels did not correlate with dengue-specific T-cell effector function (NS3-induced IFN-γ production or CD107a degranulation) or with expression of co-stimulatory/inhibitory markers including PD-1, CTLA-4, TIM-3, or CD28. Together, higher NS1 is associated with increased apoptotic susceptibility of circulating T cells without clear evidence of altered antiviral T-cell function [30].

### 3.4. NS1-Mediated Immunopathogenesis via Lipoprotein Molecules

DENV NS1 forms stable complexes with circulating lipoproteins, binding HDL with high affinity and LDL more weakly (biolayer interferometry Kd ~63 nM for HDL vs. ~1.4 μM for LDL) [32]. Structural analyses (analytical ultracentrifugation and electron microscopy) indicate that hexameric NS1 dissociates on the HDL surface into discrete NS1 dimers that insert into the hydrophobic outer layer of HDL by their hydrophobic finger-like protrusions [32,33].

Functionally, the NS1–HDL complex (but not NS1 or HDL alone) converts HDL into a pro-inflammatory stimulus for primary human monocyte-derived macrophages, increasing secretion of TNF-α, IL-6, IL-1β, and IL-10 (Luminex, 24 h) [32]. In patient samples, NS1–HDL complexes are detectable in plasma early during illness (using ApoA-I-based capture/detection ELISA formats), supporting the in vivo relevance of this circulating complex species [32]. In addition, sNS1 has structural similarities to HDL, including a central lipid cargo. Therefore, it is identified as a lipoprotein that binds to the scavenger receptor class B member 1 (SRB1) on macrophages, ECs and platelets, thereby facilitating DENV replication and contributing to severe disease manifestations such as thrombocytopenia and cytokine storms. Elevated circulating NS1 levels correlate with altered lipid profiles in dengue patients, suggesting that NS1 may compete with HDL for the SRB1 receptor [34].

Severe dengue cases have been associated with low HDL levels, which is important for removing excess cholesterol from peripheral tissues through reverse cholesterol transport (RCT). Therefore, in a macrophage model (RAW 264.7 cells), purified DENV2 NS1 increased cholesterol-rich membrane lipid rafts on non-infected cells (quantified by cholera toxin B lectin staining and flow cytometry) and enhanced subsequent DENV2 attachment (measured by qRT-PCR). These effects were associated with inflammatory activation (reflected by nitric oxide release measured by Griess assay) and were blocked by a TLR4 antagonist (LPS-RS), supporting a TLR4-dependent mechanism. Lipid-free ApoA1, the major HDL apolipoprotein, reduces this pathway by neutralising NS1-driven macrophage activation and lipid raft accumulation. Furthermore, it depleted lipid rafts via cholesterol efflux (in ABCA1-induced cells), thereby reducing DENV2 attachment. Overall, ApoA1 blunted NS1-mediated enhancement of infection and innate immune activation in vitro, consistent with a protective effect linked to RCT [35]. However, a meta-analysis of nine studies involving 1953 patients demonstrated that lower total cholesterol and LDL levels are significantly associated with increased dengue severity and risk of hypovolemic shock, whereas HDL, VLDL, and triglycerides showed no significant correlation [36].

Collectively, these studies suggest that NS1 exploits host lipid pathways. By associating with HDL (and engaging SRB1 in relevant cell types), NS1 can shift into dimeric HDL-bound forms that convert HDL into a pro-inflammatory stimulus, thereby driving cytokine release from myeloid cells. In parallel, NS1 has been shown in vitro to remodel cholesterol-rich membrane microdomains, increasing lipid rafts and facilitating viral attachment and entry (Figure 1). These mechanisms may be especially consequential in patients with severe dengue infection, potentially amplifying NS1-driven inflammatory and proviral effects observed in clinical samples and cell-based systems.

### 3.5. Contribution of Anti-NS1 Antibodies in Plasma Leakage

Anti-NS1 antibodies have been shown to function as a double-edged component of dengue-mediated immunity, with evidence supporting both protection and immunopathology. In clinical cohorts, higher circulating NS1 levels are generally associated with lower anti-NS1 titres, and NS1–antibody immune complexes have been detected early in a subset of secondary infections, consistent with antibody-mediated NS1 binding and clearance from circulation [37]. However, multiple experimental studies show pathogenic mechanisms, including immune complex-associated complement activation, endothelial cross-reactivity with downstream apoptosis, and antibody-mediated platelet opsonisation and clearance, all of which could plausibly contribute to thrombocytopenia and vascular leak.

Anti-NS1 antibodies has been shown to activate HMEC-1 through an NF-κB-regulated pathway. When HMEC-1 monolayers were exposed to anti-NS1 antibodies, increased production of IL-6, IL-8, and MCP-1, together with upregulation of ICAM-1, was demonstrated using immunostaining and flow cytometric analysis. Activation of NF-κB pathway was supported by p65 nuclear translocation and increased NF-κB DNA-binding activity. Blockade of NF-κB signalling attenuated these inflammatory responses. Functionally, anti-NS1 exposure has been shown to increase PBMC adhesion to endothelial monolayers, and neutralisation of ICAM-1 or MCP-1 reduced adhesion, supporting an MCP-1/ICAM-1-mediated leukocyte recruitment mechanism [38]. Purified anti-NS1 antibodies from dengue patients have been shown to bind HUVEC via surface protein disulfide isomerase (PDI), identified by immunoprecipitation and flow cytometric binding assays. This interaction has been shown to be associated with increased ROS generation, endothelial apoptosis, and increased permeability. In the same system, anti-NS1 antibodies have been shown to induce time- and dose-dependent HO-1 upregulation, an effect shown to be PI3K-dependent and reduced by PDI blockade, while p38 inhibition has not been shown to suppress HO-1 induction. These data have supported a coupled model in which anti-NS1–PDI engagement drives ROS-associated permeability/apoptosis while simultaneously triggering a PI3K-activated HO-1 cytoprotective response, with disease severity potentially reflecting the balance between these opposing outputs [38].

Cross-reactive anti-NS1 antibodies recognising death receptor 4 (DR4) (TRAIL-R1) have been shown to contribute to endothelial dysfunction and haemostatic disturbance. DR4-reactive immunoglobulins affinity-purified from DHF sera and from NS1-immunised rabbits were shown to increase endothelial permeability, promote apoptosis, and reduce thrombomodulin expression. In vivo, administration of DR4-reactive Ig in a sensitised “two-hit” model, where anticoagulant activity was suppressed using warfarin, exhibited plasma leakage, coagulopathy, and increased mortality. Soluble recombinant DR4 has been shown to mitigate these pathological effects, supporting DR4 as a functional target in this model [39].

Anti-NS1 antibodies have also been shown to bind and opsonise platelets, linking NS1 immunity to thrombocytopenia and coagulopathy. Patient-derived and NS1-immunised rabbits-derived anti-NS1 immunoglobulins were shown to increase platelet-bound IgM levels in proportion to anti-NS1 titres. These antibodies enhance platelet–macrophage interactions, promote complement-dependent platelet lysis, and induce abnormal platelet activation in vitro, including increased P-selectin expression, altered aggregation, and increased ATP secretion. Pre-exposure of platelets to these antibodies were shown to reduce subsequent ADP responsiveness. In mice, these antibodies cause thrombocytopenia. In warfarin-induced hypercoagulable mice, anti-NS1 antibodies further increased mortality, raised D-dimer and fibrin levels, and decreased anticoagulant proteins (protein C, protein S, and antithrombin III), resembling the coagulopathy seen in DHF [40].

Complementing this, endothelial activation by TNF-α has been shown to upregulate ICAM-1 and β3 integrin, promoting platelet adhesion. Opsonised platelets have been shown to undergo FcγR-dependent phagocytosis by activated monocytes/macrophages in reconstituted co-culture systems with monocytic THP-1 cells, cytokine-activated ECs and anti-DENV NS1 Ab-treated platelets. Blocking ICAM-1/β3 integrin or FcγR (particularly FcγRI) have been shown to reduce platelet uptake. In dengue-infected mice, anti-NS1 antibodies have been shown to increase macrophage-associated platelet clearance, supporting antibody-dependent platelet removal on activated endothelium as a plausible contributor to thrombocytopenia [41].

At the clinical level, both the magnitude and epitope specificity of the anti-NS1 response are associated with disease severity. Serial ELISA profiling during acute secondary infection has shown higher anti-NS1 titres in DHF than DF, with overlapping-peptide mapping having shown qualitatively distinct epitope-targeting patterns between DF and DHF. Furthermore, individuals with prior non-severe dengue infection have been shown to display epitope profiles resembling those observed in mild acute disease [42]. ADCC is initiated when antibodies bind infected cells, resulting in cross-linking of CD16 receptors on natural killer (NK) cells. This activation triggers NK cell degranulation and cytokine production, leading to elimination of infected cells and contributing to viral clearance. However, excessive ADCC may lead to heightened pro-inflammatory cytokine production and potential immunopathology. CD16-mediated ADCC therefore plays an important role in immune responses to DENV infection [43]. In this context, pre-existing anti-NS1 antibodies have been shown to activate NK cells through Fc receptors and mediate antibody-dependent cellular cytotoxicity (ADCC) against NS1-expressing target cells in a serotype-cross-reactive manner. This has been associated with subclinical secondary infection with higher baseline NS1 binding, stronger binding to NS1-expressing targets, and more robust NK cell activation and ADCC readouts in functional assays, supporting a protective mechanism via early clearance of NS1-expressing cells [43].

Overall, anti-NS1 immunity has been shown to comprise separable functional “modules”: (i) Fc-effector-dominant antibodies that promote NK cell ADCC and may limit systemic NS1 burden; (ii) cross-reactive antibodies that activate or injure endothelium (e.g., via NF-κB-, ROS-, or DR4/PDI-linked pathways), potentially weakening anticoagulant defences; and (iii) platelet-opsonising antibodies that promote complement/FcγR-dependent platelet injury and clearance (Figure 1). Framing the literature through these modules can help reconcile why anti-NS1 responses have been shown to be protective in some contexts yet pathogenic in others, depending on antibody specificity, Fc functionality, host target cross-reactivity, and timing relative to the critical phase.

## 4. NS1-Based Vaccine Development

Developing a safe tetravalent dengue vaccine remains challenging due to incomplete understanding of dengue immunopathogenesis and the risk of worsening disease. The first-generation vaccine, Dengvaxia, targets only structural proteins (prM/E), resulting in uneven serotype protection and no immunity against NS1, one of the key mediators of vascular leak and severe disease. This focus leads to uneven protection across the four different Dengue types and, more importantly, fails to trigger immunity against the NS1 protein. Without targeting NS1, the vaccine cannot block the viral toxin effects that lead to severe symptoms like internal bleeding and vascular leak [44].

Second-generation vaccines like live-attenuated TAK-003 vaccine induce broader immunity, including T-cell responses against structural and non-structural proteins, and help preserve endothelial integrity regardless of prior exposure [44]. However, limitations remain, including variable efficacy across serotypes (notably weaker protection against DENV-3 in seronegative individuals), potential safety concerns (genetic reversion to wild-type virulence, possible mosquito transmission of vaccine virus and the risk of antibody-dependent enhancement (ADE)), and uncertainty in predicting neutralising antibody titers for actual protection, underscoring the need for continued long-term monitoring to fully define the safety and durability of the vaccine [45].

Incorporation of NS1 into next-generation platforms (TAK-003 refinement, mRNA vaccines) offers a promising strategy by inducing both antitoxin (humoral) and cell-mediated immunity while minimising harmful cross-reactivity through epitope engineering. NS1-based tetravalent vaccines may represent a key direction toward safer and more effective dengue prevention.

## 5. Conclusions

DENV NS1 contributes to dengue-related immunopathogenesis through multiple intersecting pathways. It directly compromises endothelial barrier function by promoting glycocalyx degradation and destabilising junctional integrity, while also modulating complement to blunt antiviral effector mechanisms. NS1 additionally engages lipid pathways and receptor interactions that can amplify inflammatory signalling and facilitate viral entry, and it conditions innate immune cells toward exaggerated cytokine responses. Anti-NS1 antibodies appear similarly bifunctional: pathogenic subsets cross-react with host targets, promoting endothelial damage, increased permeability, platelet clearance, and thrombocytopenia, whereas protective antibodies preferentially drive Fc-mediated NK cell ADCC and may support cross-serotype clearance of NS1-expressing cells. Clinically, higher circulating NS1, MIF, and heparanase levels correlate with severe dengue infection, including vascular leak and coagulopathic features. Together, these data position NS1 as a central molecular bridge between immune dysregulation and vascular barrier failure, helping explain progression from uncomplicated dengue infection to life-threatening disease while highlighting NS1-linked pathways as rational therapeutic targets.

## Data Availability

All data supporting the conclusions of this article are included within the article and its reference sources.

## Abbreviations

ADCC	Antibody-dependent cellular cytotoxicity
ADP	Adenosine diphosphate
Ang-2	Angiopoietin-2
DR4	Death receptor 4
DF	Dengue fever
DHF	Dengue haemorrhagic fever
DSS	Dengue shock syndrome
DENV	Dengue virus
PDI	Protein disulfide isomerase
EGL	Endothelial glycocalyx layer
GSK-3β	Glycogen synthase kinase-3 beta
GAG	Glycosaminoglycans
HDL	High-density lipoprotein
HSP27	Heat shock protein 27
HPA-1	Heparanase-1
HO	Heam oxygenase
HMEC	Human microvascular endothelial cells
HPMEC	Human pulmonary microvascular endothelial cells
HUVEC	Human umbilical vein endothelial cells
LDL	Low-density lipoprotein
LPS	Lipopolysaccharide
MAC	Membrane attack complex
MIF	Macrophage migration inhibitory factor
MMP-9	Matrix metalloproteinase-9
NF-κB	Nuclear factor kappa B
NK	Natural killer cells
NS1	Non-structural protein 1
p38 MAPK	p38 mitogen-activated protein kinase
PAMP	Pathogen-associated molecular patterns
PRR	Pattern-recognition receptors
PBMC	Peripheral blood mononuclear cells
qRT-PCR	Quantitative reverse transcription polymerase chain reaction
RCT	Reversing cholesterol transport
RhoA/ROCK	RhoA/Rho-associated coiled-coil-containing protein kinase
ROS	Reactive oxygen species
SRB1	Scavenger receptor class B, member 1
SVP	Saphenous vein pericytes
sNS1	Soluble NS1
TCC	Terminal complement complex
TLR4	Toll-like receptor-4
TEER	Trans-endothelial electrical resistance
ZO	Zona-occludens
